# Genetic variation of *Plasmodium falciparum* histidine-rich protein 2 and 3 in Assosa zone, Ethiopia: its impact on the performance of malaria rapid diagnostic tests

**DOI:** 10.1186/s12936-021-03928-3

**Published:** 2021-10-09

**Authors:** Gezahegn Solomon Alemayehu, Alebachew Messele, Kayla Blackburn, Karen Lopez, Eugenia Lo, Daniel Janies, Lemu Golassa

**Affiliations:** 1 Addis Ababa University, Aklilu Lemma Institute of Pathobiology, Addis Ababa, Ethiopia; 2 Departments of Bioinformatics and Genomics, University of North Carolina at Charlotte, Charlotte, NC 28223 USA; 3 Department of Biological Sciences, University of North Carolina at Charlotte, Charlotte, NC 28223 USA; 4 School of Data Sciences, University of North Carolina at Charlotte, Charlotte, NC 28223 USA

**Keywords:** RDT, *Plasmodium falciparum*, Genetic variation, PfHRP2, PfHRP3, Amino acid sequence, Repeat type, Assosa, Ethiopia

## Abstract

**Background:**

Rapid diagnostic tests (RDT) are commonly used for the diagnosis of malaria caused by *Plasmodium falciparum*. However, false negative results of RDT caused by genetic variation of *P. falciparum* histidine-rich protein 2 and 3 genes (*pfhrp2/3)* threaten existing malaria case management and control efforts. The main objective of this study was to investigate the genetic variations of the *pfhrp2/3* genes.

**Methods:**

A cross-sectional study was conducted from malaria symptomatic individuals in 2018 in Assosa zone, Ethiopia. Finger-prick blood samples were collected for RDT and microscopic examination of thick and thin blood films. Dried blood spots (DBS) were used for genomic parasite DNA extraction and molecular detection. Amplification of parasite DNA was made by quantitative PCR. DNA amplicons of *pfhrp2/3* were purified and sequenced.

**Results:**

The PfHRP2 amino acid repeat type isolates were less conserved compared to the PfHRP3 repeat type. Eleven and eight previously characterized PfHRP2 and PfHRP3 amino acid repeat types were identified, respectively. Type 1, 4 and 7 repeats were shared by PfHRP2 and PfHRP3 proteins. Type 2 repeats were found only in PfHRP2, while types 16 and 17 were found only in PfHRP3 with a high frequency in all isolates. 18 novel repeat types were found in PfHRP2 and 13 novel repeat types were found in PfHRP3 in single or multiple copies per isolate. The positivity rate for PfHRP2 RDT was high, 82.9% in PfHRP2 and 84.3% in PfHRP3 sequence isolates at parasitaemia levels > 250 parasites/µl. Using the Baker model, 100% of the isolates in group A (If product of types 2 × type 7 repeats ≥ 100) and 73.7% of the isolates in group B (If product of types 2 × type 7 repeats 50–99) were predicted to be detected by PfHRP2 RDT at parasitaemia level > 250 parasite/μl.

**Conclusion:**

The findings of this study indicate the presence of different PfHRP2 and PfHRP3 amino acid repeat including novel repeats in *P. falciparum* from Ethiopia. These results indicate that there is a need to closely monitor the performance of PfHRP2 RDT associated with the genetic variation of the *pfhrp2* and *pfhrp3* gene in *P. falciparum* isolates at the country-wide level.

**Supplementary Information:**

The online version contains supplementary material available at 10.1186/s12936-021-03928-3.

## Background

Malaria caused by *Plasmodium falciparum* has remained a life-threatening infectious disease despite significant advances made towards malaria control and elimination in most malaria endemic countries in the world [1]. *Plasmodium falciparum* is the most common species that causes complicated malaria in humans. The genetic variation in the genome of *P. falciparum* has the potential to make pathogenesis virulent and challenge the accuracy of diagnosis [2]. One of the strategic pillars to reduce the morbidity and mortality associated with falciparum malaria is the provision of rapid and accurate malaria diagnostic tools in malaria-endemic settings [3].

Rapid diagnostic tests (RDTs) are widely and routinely used in remote and resource limited settings. Currently available malaria RDTs utilize parasite-specific antigens produced by malaria parasites for diagnosis [4]. The most commonly used biomarkers in antigen-based malaria RDTs are lactate dehydrogenase (LDH), aldolase, and histidine-rich protein 2 (HRP2) [5, 6]. The PfHRP3 antigen, also produced by *P. falciparum,* has been shown to share high homology with PfHRP2 [7] and plays a substantial role in the detection of *P. falciparum* infections by PfHRP2-based RDTs [8].

The *pfhrp2* and *pfhrp3* genes are located in the subtelomeric region of chromosome 8 and 13, respectively. Both *pfhrp2* and *pfhrp3* genes consist of a single intron and two exons [7, 9]. Exon 2 in both *pfhrp2* and *pfhrp3* encodes amino acids, which exhibit high levels of homology, suggesting that these genes were originated by duplication and divergence from a common ancestral sequence [7, 10]. The amino acid sequences of PfHRP2 and PfHRP3 contain several tandem copies of alanine and histidine-rich repeat. The PfHRP2/3 amino acid sequences with multiple repeats of AHH and AHHAAD contain approximately 35% histidine (H), 40% alanine (A), and 12% aspartate (D) [7]. Baker et al. showed several epitopes in PfHRP2 and PfHRP3 such as type 2 (AHHAHHAAD), type 4 (AHH), and type 7(AHH AAD) that are targeted by monoclonal antibodies against PfHRP2 and PfHRP3 antigens based on the PfHRP2 RDTs [11, 12].

However, frequent recombinations that occur at the subtelomeric regions contribute to vast genetic variation in the *Pfhrp2* and *pfhrp3* genes [8, 13, 14]. Recent studies from several African and South American countries showed that extensive variation in the amino acid sequences of PfHRP2 and PfHRP3 could influence the frequency of the respective epitopes recognized by monoclonal antibodies [8, 12, 15]. Furthermore, deletions of the *pfhrp*2/3 gene cause a lack of target antigen in the diagnosis of *P. falciparum* malaria [16–18]. Genetic variation and deletion of PfHRP2 and PfHRP3 amino acid sequence cause false negative testing using PfHRP2-based RDTs [19, 20]. Ultimately, inaccurate diagnosis threatens ongoing efforts in malaria control and prevention [21].

Before this study, there were no data on the genetic variation of the *pfhrp2/3* gene in Assosa zone, northwest Ethiopia. Information on the abundance of PfHRP2 and PfHRP3 peptides repeats in *P. falciparum* isolates from geographically distinct regions is vital to monitor the diagnostic performance of PfHRP2 RDTs. Thus, the aim of this study was to determine the extent of genetic variation of *pfhrp2* and *pfhrp3* in clinical isolates, where *P. falciparum* is highly prevalent and PfHRP2 RDT is used for front-line diagnosis.

## Methods

### Study area and period

This study was carried out during the low and high transmission seasons in four selected health facilities: Assosa, Bambasi, Kurmuk and Sherkole Health Centres in Assosa Zone, Benishangul-Gumuz regional state, northwest Ethiopia, April to December 2018. The study area is a high malaria transmission setting. The Assosa Zone is located on the border of Sudan, where transboundary transmission of malaria likely occurs. The map of the study area is indicated in Additional file 1.

### Study design, sample size and sampling technique

A cross-sectional health facilities based study was conducted to assess genetic variation of *pfhrp2/3* in clinical isolates. The study populations were residents of the Assosa Zone which includes all patients with clinical suspicion of malaria aged ≥ 5 years in the selected health facilities during the study period. The inclusion criteria were all study participants with clinical suspicion of malaria who gave their written consent and/or assent. High-quality sequence data were included for molecular analysis of *pfhrp*2/3 genetic variation. Study subjects who worked or lived outside the Assosa Zone catchment area and or who were unwilling to participate were excluded from the study. Data with poor quality sequences were also excluded.

The sample size was calculated based on the single population proportion formula n = z^2^p (1 − p)/d^2^ [22]; Where, n = the sample size, z = 1.96 at 95% confidence interval (CI), d = margin of error at 5%, p (expected malaria prevalence rate) is 40% prevalence of symptomatic malaria in a hospital study of the region [23]. As a result, the sample size calculated with 10% non-response was 406 study participants. A total of 812 study participants were involved in this study, 406 study participants in low and 406 study participants in high transmission seasons.

The study area, Assosa Health Centre, Bambasi Health Centre, Kurmuk Health Centre and Sherkole Health Centre, was selected using a simple random sampling technique among eight districts in the Assosa zone. Then, allocation of the study participants to each selected health centre was performed based on proportion of confirmed malaria case in each selected district/Woreda.

### Sample collection

**Fig. 1:**
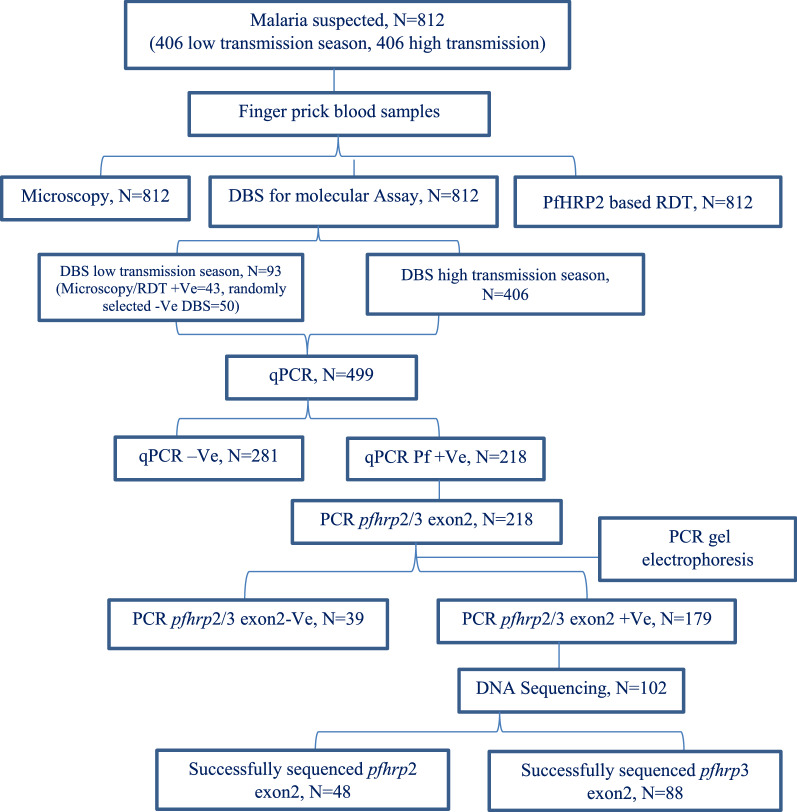
Study flow chart for molecular analysis of *pfhrp2/3* genetic variation. DBS: Dried blood Spot, qPCR: quantitative PCR, Pf: *P. falciparum*, −Ve: negative, + Ve: Positive, N: number of samples,

In a single finger-prick, capillary blood samples were collected from malaria suspected individuals for microscopy, malaria RDTs as well as dried blood spots (DBSs) for molecular assay. Overall work flow of this study indicated in Fig. 1.

### Microscopy and malaria RDT

The CareStart™ malaria RDTs (Pf/PV HRP2/PLDH) were used following manufacturer’s instructions. Thick and thin blood smears were stained with a 10% buffer-diluted working solution of Giemsa for microscopic detection and the measurement of parasite density according to World Health Organization recommendations [24].

### Parasite DNA extraction and molecular analysis

Genomic DNA was extracted from DBSs using the chelex-saponin method as described previously [25]. *P. falciparum* identification was confirmed by SYBR Green quantitative PCR (qPCR) assay after amplification of DNA coding for 18S ribosomal RNA using species-specific primers [26]. After confirming *P. falciparum* positive samples, all PCR reaction of *pfhrp2* exon2 and *pfhrp*3 exon2 were performed at 25 µl total with 2 × Promega Hot Start Master Mix (Promega Corporation, Madison, USA), 0.4 µl each of forward and reverse primers, and 3 µl of extracted template DNA using PCR conditions previously described [17, 21, 27]. PCR products of *pfhrp2 exon2* and *pfhrp3 exon2* were separated by electrophoresis on a 2% agarose gel, visualized in a UV transilluminator, and the size of the expected amplicons compared to 1 kb DNA ladder. Primer sequences, PCR conditions, and expected amplicon size of *pfhrp2* exon2 and *pfhrp3* exon2 are provided in Additional file 2.

### Sequencing analyses

A total of 48 *pfhrp2 exon 2* and 88 *pfhrp3 exon 2* high quality sequence data were included for molecular analysis of *pfhrp2/3* genetic variation. *Pfhrp*2/3 sequence of this study deposited in the NCBI Gen Bank database (with Accession number: MZ050658-MZ050792). All amplicons were cleaned using Exosap and sequenced using Sanger technology with ABI BigDyeTM Terminator v 3.1 chemistry (Thermofisher, Santa Clara, CA) and ran on a 3130 Genetic Analyzer (Thermofisher, Santa Clara, CA). Sequences were then cleaned and analysed using CodonCode Aligner Program V.6.0.2 (CodonCode Corporation, Centerville, MA). Nucleotide sequences were inputted into the ExPASy Translate Tool (Swiss Institute of Bioinformatics Resource Portal) and translated into corresponding amino acids sequence using the correct open reading frame. The amino acid repeat sequences in *pfhrp2* and *pfhrp3* were given a numeric code in the system as described previously [8, 11].

### Data analysis

The proportions of each amino acid repeat of PfHRP2 and PfHRP3 in the *P. falciparum* isolates were analysed using Statistical Package for Social Sciences (SPSS) version 20. The association between PfHRP2 RDT results (sequenced samples could be RDT positive or negative) and certain group of amino acid repeat was tested by Chi square and Fisher’s exact test. P-values < 0.05 were interpreted as statistically significant. Bakers’ model [8, 11] used for the prediction of PfHRP2 RDT sensitivity, the sequences of PfHRP2 were classified into four groups based on combined length of types 2 × type 7 repeats. They include group A (≥ 100, very sensitive), group B (50–99, sensitive), group C (< 43, non-sensitive) and group I (44–49, borderline sensitive). The PfHRP2/3 amino acid sequences obtained from Ethiopia were compared with isolates from other countries based on the sequences deposited in the National Center for Biotechnology Information’s (NCBI) GenBank using Basic Local Alignment Search Tool for Protein analysis (BLASTP).

## Results

### Genetic variation of PfHRP2 and PfHRP3 amino acids repeats

Among the 48 PfHRP2 sequenced samples, the length of *pfhrp2* exon 2 sequences varied between 453 and 873 base pairs (bp) in DNA and 150 to 290 residues in amino acids (aa). A total of 11 known PfHRP2 amino acid repeat types were identified (Table 1).

**Table 1:** Base pair length, amino acid length, number of known repeat type, and novel variants of PfHRP2 and PfHRP3 by study sites

District	PfHRP2					PfHRP3				
	Total No			Mean		Total No			Mean	
	Sequence analyzed	Known repeat type	Novel variants	Base pair length (range)	Amino acid length(range)	Sequence analyzed	Known repeat type	Novel variants	Base pair length (range)	Amino acid length(range)
Sherkole	24	11	14	542 (453–825)	239 (150–283)	34	8	7	555 (466–636)	182 (154–211)
Bambasi	15	11	2	735 (554–873)	244 (184–289)	32	8	5	593 (493–646)	196 (164–215)
Kurmuk	7	10	2	733 (638–872)	244 (212–290)	20	8	7	573 (461–654)	190 (153–217)
Assosa	2	9	1	787 (770–805)	230 (212–247)	2	8	0	515 (464–567)	171 (154–188)

**Fig. 2:**
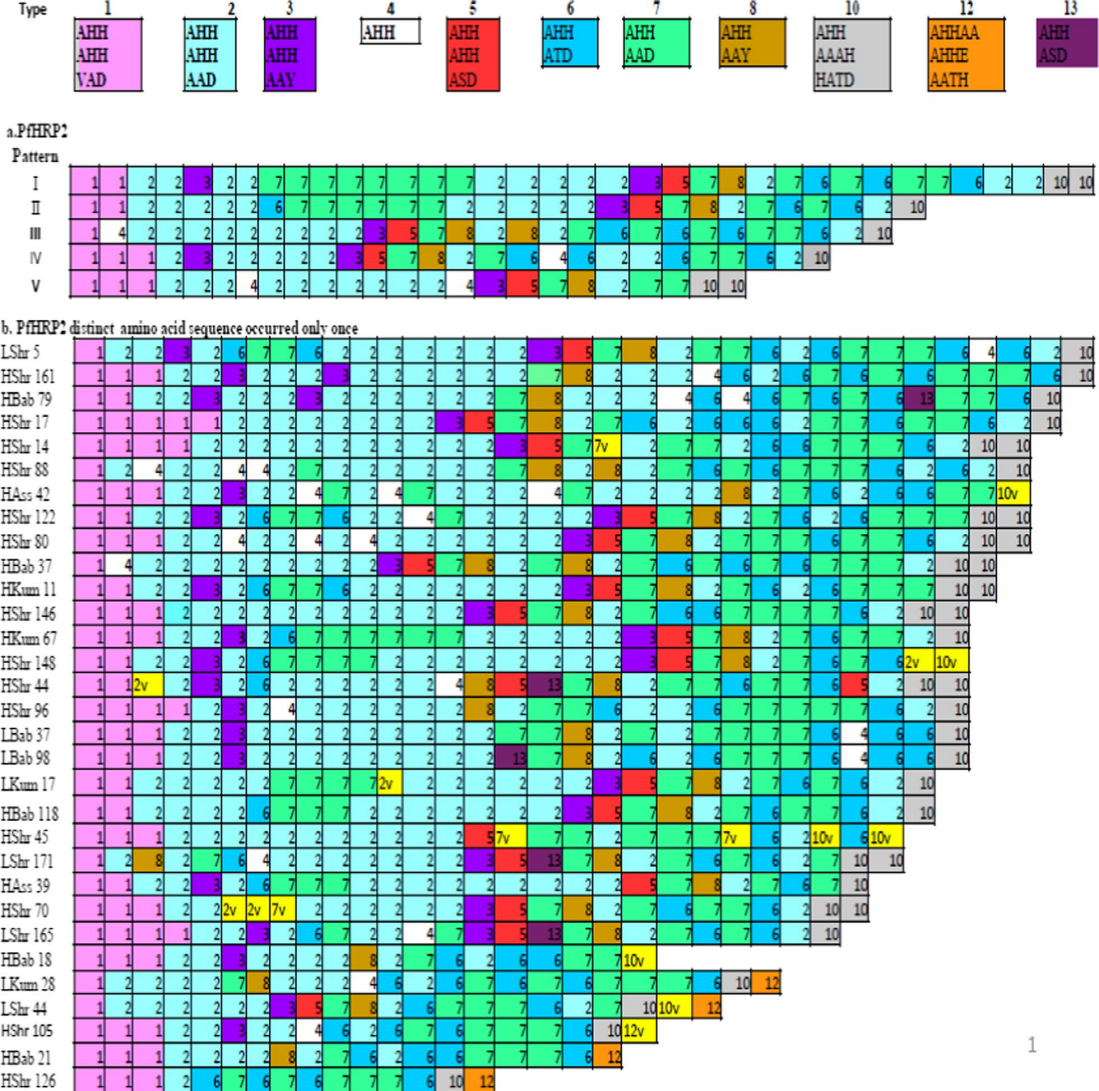
PfHRP2 structural organization in clinical isolates collected from Assosa Zone, Ethiopia. **a** PfHRP2 patterns: Pattern I, IV & V in two isolates; Pattern III in three isolates; Pattern II among eight isolates. **b** Thirty-one distinct PfHRP2 amino acid sequence occurred only once with different sample code letter (HShr, Lshr, HBab, LBab, Hkum, Lkum, HAss and LAss). A total of 11 PfHRP2 amino acid repeat type is differentiated by colour block with their respective repeat type number. Unique variant repeat (V) display by yellow colour

Nearly 65% (31/48) of the distinct PfHRP2 amino acid sequence occurred only once, while five PfHRP2 amino acid sequence patterns (I–V) were found in more than one isolate (Fig. 2). The structural organizations of the amino acid repeats were found at different positions of the PfHRP2 amino acid sequences. PfHRP2 amino acid sequences of all the isolates started with a type 1 repeat. 89.6% of the PfHRP2 amino acid sequences ended with a type 10 repeat (occurred in five PfHRP2 patterns and distinct PfHRP2 sequence). 10.4% of the PfHRP2 amino acid sequences contained a type 12 repeat (occurred only in four distinct PfHRP2 isolates). About 48% (23/48) of the isolates had a PfHRP2 repeat motif composed of types 2, 3, 5, 7, 8, 2, and 7, and this motif was absent in all PfHRP2 patterns. About 15% (7/48) of the isolates had a PfHRP2 repeat motif composed of types 7, 8, 2, and 7, and this motif was found in all PfHRP2 patterns except pattern III (Fig. 2). Types 2 and 7 were the most frequent repeats among isolates and were broadly distributed in PfHRP2 amino acid sequence.

The type and frequency of each PfHRP2 amino acid repeat varied among parasite isolates at study sites. Each PfHRP2 amino acid sequence contains 15–36 repeats. Repeat types 1, 2 and 7 were found in 100% of the isolates, repeat types 6 and 10 were found in 95.8% of the isolates, and type 5, type 3 and type 8 were found in 75–91.7% of the isolates. Lower frequencies of PfHRP2 amino acid repeats were observed in type 4 (found in 20 isolates; 41.7%), as well as types 12 and 13 (found in 5 isolates; 10.4%). By contrast, types 9 and 11 were not identified in any PfHRP2 amino acid sequences in this study (Table 2).

**Table 2:** Distribution of PfHRP2 and PfHRP3 Amino acids repeat in *P. falciparum* isolates in Assosa zone, Northwest Ethiopia

Type of repeat	Amino acid repeat sequence*	PfHRP2 (n = 48)		PfHRP3 (n = 88)	
		No (%)	Min to Max number	No (%)	Min to Max number
1	AHHAHHVAD	48 (100)	1–5	83 (94.3)	0–3
2	AHHAHHAAD	48 (100)	1–15	0 (0.0)	0
3	AHHAHHAAY	42 (87.5)	0–2	0 (0.0)	0
4	AHH	20 (41.7)	0–3	78 (88.6)	0–1
5	AHHAHHASD	36 (75.0)	0–1	0 (0.0)	0
6	AHHATD	46 (95.8)	0–6	0 (0.0)	0
7	AHHAAD	48 (100)	3–13	85 (96.6)	0–2
8	AHHAAY	44 (91.7)	0–2	0 (0.0)	0
9	AAY	0 (0.0)	0–0	0 (0.0)	0
10	AHHAAAHHATD	46 (95.8)	0–2	0 (0.0)	0
11	AHN	0 (0.0)	0–0	0 (0.0)	0
12	AHHAAAHHEAATH	5 (10.4)	0–1	0 (0.0)	0
13	AHHASD	5 (10.4)	0–1	0 (0.0)	0
14	AHHAHHATD	0 (0.0)	0	0 (0.0)	0
15	AHHAHHAAN	0 (0.0)	0–0	84 (95.5)	0–2
16	AHHAAN	0 (0.0)	0–0	88 (100)	7–16
17	AHHDG	0 (0.0)	0–0	88 (100)	1–8
18	AHHDD	0 (0.0)	0–0	87 (98.9)	0–4
19	AHHAA	0 (0.0)	0–0	0 (0.0)	0
20	SHHDD	0 (0.0)	0–0	87 (98.9)	0–2
21	AHHAHHATY	0 (0.0)	0–0	0 (0.0)	0
22	AHHAHHAGD	0 (0.0)	0–0	0 (0.0)	0
23	ARHAAD	0 (0.0)	0–0	0 (0.0)	0
24	AHHTHHAAD	0 (0.0)	0–0	0 (0.0)	0–0

*PfHRP2 and PfHRP3 amino acid repeat motif as describe by Baker et al. [11]

N: Total number of PfHRP2 and PfHRP3 amino acid sequence in *P. falciparum* isolates

Min: Minimum number of amino acid repeat, Max: maximum number of amino acid repeat

Almost all PfHRP2 repeat types were found in all study sites with a slight difference in the frequency of repeat type within and between the study sites. Repeat types 1, 2 and 7 were found in all isolates of Sherkole, Bambasi, Kurmuk, and Assosa. Repeat types 3, 5, 6, 8 and 10 were found in 66–100% isolates among study sites. Repeat types 4 and 12 occurred in 6.7–62.5% of the isolates among study sites. Type 13 was found only in Sherkole (12.5%; 3/24) and Bambasi (13.3%; 2/15) (Additional file 3).

**Fig. 3:**
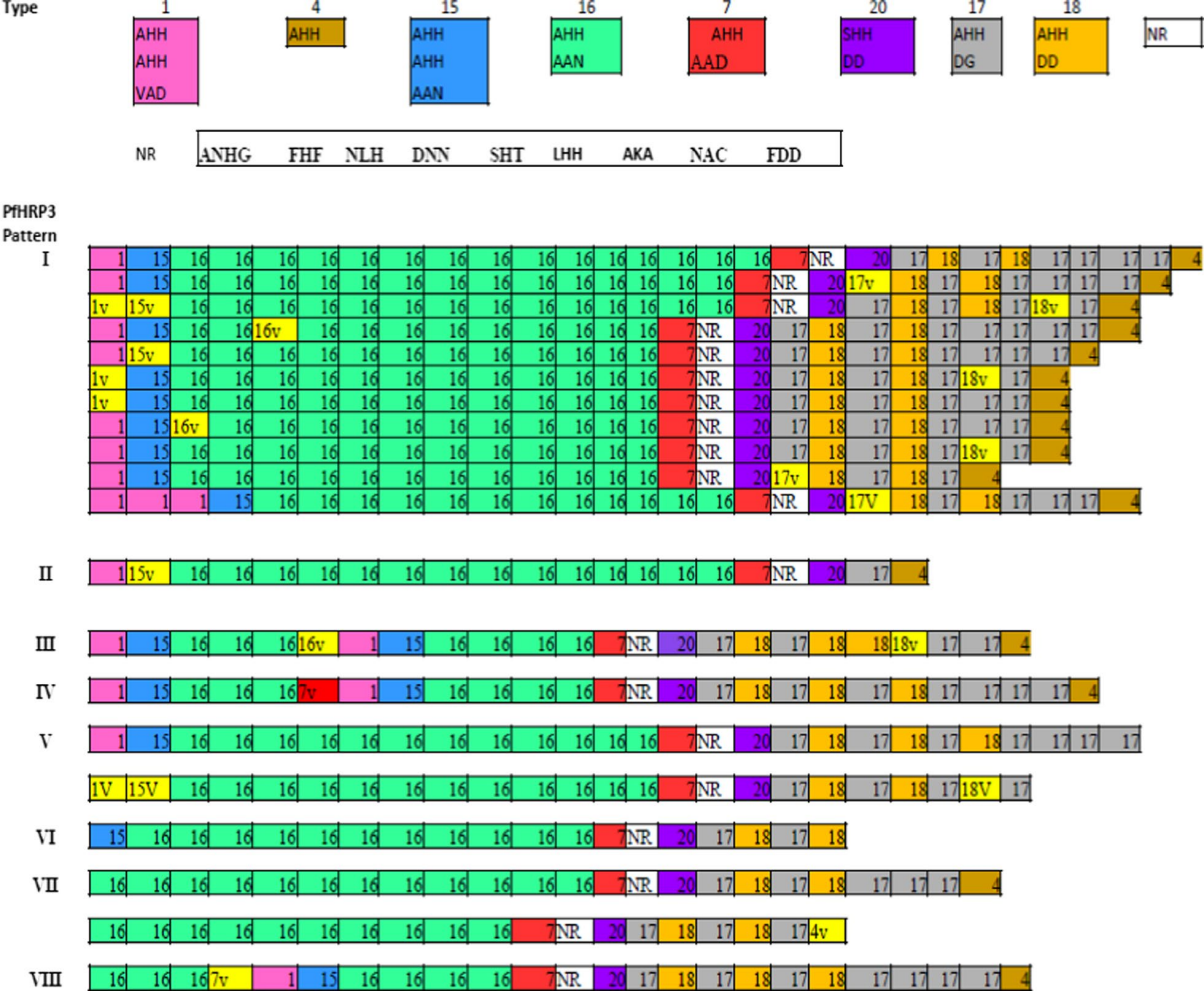
PfHRP3 structural organization in Assosa isolates. Eight PfHRP3 patterns identified in more than one isolates. A total of eight PfHRP3 amino acid repeat type is differentiated by color block with their respective repeat type number. Unique variant repeat (V) display by yellow color. NR: Non-repetitive region

Among the 88 sequenced samples, the length of the *pfhrp3 exon 2* ranged from 461 to 654 bp in DNA and 153 to 217 residues in amino acids. Of 88 PfHRP3 sequence isolates, eight different PfHRP2 amino acid repeat types were identified (Table 1). Eight distinct PfHRP3 patterns detected in more than one isolate (Fig. 3). The PfHRP3 sequences began with type 1 (present in PfHRP3 pattern I–V), type 16 (present in PfHRP3 pattern VII–VIII), and type 15 (present in PfHRP3 pattern VI) repeats in 93.2%, 4.6%, and 2.3% of the isolates, respectively. The PfHRP3 sequences ended with type 4 (present in PfHRP3 pattern I–IV and VII–VIII), type 17 (present in PfHRP3 pattern V), and type 18 (present in PfHRP3 pattern VI) repeats in 87.5%, 11.4%, and 1.1% of the isolates, respectively.

All isolates of the PfHRP3 amino acid sequence in this study contained a non-repeating sequence (ANHGFHFNLHDNNSHTLHHAKANACFDD) between types 7 and 20. Most of the isolates (96.6%; 85/88) had a similar unique PfHRP3 repeat motif with structural organization of repeat types 15, 16, 20, 17, and 18, and this motif was found in all PfHRP3 patterns except type VII (Fig. 3).

The total number of amino acid repeats and each repeat within the PfHRP3 sequence varied among the parasite isolates. Each PfHRP3 amino acid sequence consisted of 18–28 repeats. Repeats types 16 and 17 were found in 100% of the isolates. Types 1, 4, 7, 15, 18, and 20 were detected in more than 88% of the isolates. In contrast, types 2–3, 5–6, 8–9, 10–14, and 19 were not identified in the PfHRP3 amino acid sequences in this study. All PfHRP3 repeat types were found in all study sites with some difference in the frequency of the repeat type within and between study sites. Repeat types 16, 17, 18, and 20 were found in 100% of the isolates in Sherkole, Bambasi, Kurmuk and Assosa. Types 18 and 20 in 95% of the isolates in Kurmuk. On the other hand, repeat types 1, 4, 7, and 15 were found in 84.4–100% of the isolates among study sites (Additional file 3).

### Analysis of Ethiopian PfHRP2/3 amino acid sequences against the global isolates

BLAST analysis of PfHRP2/3 amino acid sequences were performed for each of the sequences obtained from Ethiopia to look for sequence similarities with other countries available from NCBI. PfHRP2 protein BLAST (BLASTP) analysis reveals a range of 88.50–98.82% similarity with *P. falciparum* isolates from Africa, India, and Myanmar, accession numbers from similar sequences are indicated in Additional file 4. Likewise, the BLASTP analysis of PfHRP3 reveals a range of 93.55–100% similarity with *P. falciparum* isolates from Kenya and India, accession numbers from similar sequences are indicated in Additional file 5.

### Comparison of novel variants of PfHRP2/3 repeat types in Ethiopia with global diversity

A total of 18 novel variants of PfHRP2 repeat types were found in this study (Table 3). Of these novel PfHRP2 amino acid repeat sequences, 15 were not reported previously. Though type 7 (AHH**D**D) and type 10 (AHHAA**T**HHATD, AHHAAAHHA**N**D) repeats were reported previously (Additional file 6), they occurred at a very low frequency. Similarly, 13 new PfHRP3 repeat types were identified among the Ethiopian isolates. Among them, 12 were not previously reported. Only type 20 (SHHD**G**) was previously reported (Additional file 7) and it occurred at high frequency among the Ethiopian isolates.

**Table 3:** List of Novel repeat /variants of PfHRP2 and PfHRP3 amino acid repeat types

Type of repeat	Known amino acid repeat	PfHRP2		PfHRP3	
		Novel repeat	Repeat frequency	Novel repeat	Repeat frequency
1	AHHAHHVAD	–	–	**D**HHAHHVAD	1
				**P**HHAHHVAD	4
				**SP**HAHHVAD	1
2	AHHAHHAAD	AHHAHH**E**AD	1		
		AHHA**Q**HAAD	1		
		AHHAHHA**HH**	1		
		A**PD**HHAHH	1		
		AHHAH**V**AAD	1		
		AHHAHHAA**Y**	1		
3	AHHAHHAAY	AHHAH**Y**AAY	1		
4	AHH			**V**HH	1
7	AHHAAD	AHHA**VH**	1		
		AHHA**D**D	2		
		AHHA**V**D	1		
10	AHHAAAHHATD	AHHAAAHHA^†^	1		
		AHHAAA**D**HHATD	1		
		AHHAAAH**Q**ATD	1		
		AHHAAAHHA**N**D	1		
		AHHAAAHHA**R**D	1		
		AHHAA**D**AHHATD	1		
		AHHAA**T**HHATD	1		
12	AHHAAAHHEAATH	**V**HHAAAHHEA**PLC**	1		
15	AHHAHHAAN	**–**		AHHAHHA**D**N	3
		**–**		AHHAHH**V**AN	1
16	AHHAAN	**–**		AHHA**GD**	4
		**–**		A**LL**AAN	1
		**–**		**S**HHAAN	1
18	AHHDD			**E**HHD**E**	1
				AHHD**E**	3
				A**R**HDD	1
20	SHHDD			SHHD**G**	7

The asterisks (†) show deletion position of amino acid code (double letter), bold underline letter indicate position of novel repeat with replacement of one or more amino acid compared to known repeat

### Effect of genetic variation of pfhrp2/3 on performance of RDTs

Of the 48 *P. falciparum* samples included in *Pfhrp2* exon2 sequence analysis, 72.9% (35/48) were detected as positive by PfHRP2 RDT. Among these PfHRP2 RDT positive samples, 82.9% (29/35) was found with a parasitaemia level > 250 parasite /μL. However, all PfHRP2 RDT negative samples (13/48) were submicroscopic (Additional file 8). Similarly, among 88 *P. falciparum* samples included in *Pfhrp3* exon2 sequence analysis, 79.5% (70/88) was detected by PfHRP2 RDT and 84.3% (59/70) of these positive samples had parasitaemia levels > 250 parasites/µl (Additional file 9).

The performance of PfHRP2 RDT and the number of PfHRP2 amino acid repeat 1 (P = 0.944), repeat 2 (P = 0.456), repeat 6 (P = 0.95), repeat 7 (P = 0.486), and repeat 10 (P = 0.273) observed in the samples were not statistically associated with one another. Likewise, the performance of PfHRP2 RDT and variation of PfHRP3 amino acid repeat 1 (P = 0.928), repeat 4 (P > 0.05), repeat 7 (P > 0.05), repeat 15 (P > 0.05), repeat 18 (P = 0.436), and repeat 20 (P > 0.05) were not statistically associated. Interestingly, significant association was detected between performance of PfHRP2 and the number of PfHRP3 repeat 16 (P = 0.031) and repeat 17 (P < 0.001) (Additional file 10).

Amino acid changes in PfHRP2 and PfHRP3 repeat types observed in this study might affect the performance of PfHRP2 RDT. Eighteen novel PfHRP2 and 13 novel PfHRP3 repeat types were identified within 13 PfHRP2 and 24 PfHRP3 sequence in the *P. falciparum* population, respectively. Of 13 PfHRP2 sequences, 38.5% (5/13) that had the novel variants were detected negative by PfHRP2 RDT (Additional file 8). Of the 24 PfHRP3 sequences, 37.5% (9/24) that had the novel variants were negative by PfHRP2 RDT (Additional file 9).

### Prediction of PfHRP2 RDT sensitivity using Bakers’ model

According to the Baker's model, most of the isolates in this study was from group B (54.2%, 26/48) followed by group A (22.9%, 11/48). Among these isolates, 100% (8/8) in group A and 73.7% (14/19) in group B were predicted to be detected by PfHRP2 RDT at parasitaemia level > 250 parasite/μl. About 86% (6/7) of *P. falciparum* isolates in group C were sensitive to PfHRP2 RDT at parasitaemia level > 250 parasites /μl in this study (Table 4).

**Table 4:** Frequency of PfHRP2 sequence groups based on the Baker’s model and PfHRP2 RDT results (n = 48)

Group	Frequency (N = 48)	Prediction of PfHRP2 RDT Positivity	Microscopy > 250 parasite/µl
	No (%)	No (%)	No (%)
A	11 (22.9)	8 (72.7)	8 (100)
B	26 (54.2)	19 (73.1)	14 (73.7)
C	10 (20.8)	7 (70.0)	6 (85.7)
I	1 (2.1)	1 (100)	1 (100)

PfHRP2 sequence isolates were classified into four groups according to the frequency number of types 2 × type 7 repeats: group A (≥ 100, very sensitive), group B (50–99, sensitive), group C (< 43, non-sensitive) and group I (44–49, borderline) as describe previously (Baker et al. [8])

## Discussion

The present study provides valuable information on genetic variations in the PfHRP2 and PfHRP3 amino acid repeat types, which could affect the performance of PfHRP2 RDT for *P. falciparum* diagnosis*.* The structural organization of PfHRP2 amino acid sequences were less conserved than PfHRP3 among the Ethiopia isolates. The sequence length and the number of amino acid repeat types of PfHRP2 and PfHRP3 in this study were comparable to those previously reported in Yemen [28] and Madagascar [15], but lower than those in Kenya [29] and Ghana [30] and higher than in India [31].

All PfHRP2 repeat types started with repeat type 1 in the Ethiopian *P. falciparum* isolates, similar to previous reports in Kenya [29], Yemen [28], Senegal [32], and outside Africa [8]. The majorities of the PfHRP2 repeat ended with type 10 repeat which showed discordance with the previous reports from Africa [28, 29, 32] and Asia [31]. On the other hand, a small proportion of the isolates (10.4%) ended with a type 12 PfHRP2 repeats coincide with reports from Senegal [33]. Two types of PfHRP2 repeat motifs structural organization repeat motifs (2, 3, 5, 7, 8, 2, and 7) and (7, 8, 2, 7), were identified [8, 29].

Eleven different PfHRP2 repeats and eight different PfHRP3 repeats were found at Sherkole, Bambasi, Kurmuk and Assosa with slight difference in the frequency of the repeat type within and between *P. falciparum* isolates in Ethiopia, similar to those reported in Africa [29, 30] and worldwide [8].The PfHRP2 repeat types observed in the present study were previously reported in Africa [28–30], Asia [8, 31], and America [34, 35]. BLAST analysis of the Ethiopia PfHRP2 and PfHRP3 amino acid sequences revealed the presence of similarities and shared identity with isolates from Kenya. Interestingly, the amino acid sequences similarities of PfHRP2 and PfHRP3 also extended beyond the border of Ethiopia, India, and Myanmar, which is also in agreement with a recent study from Ghana [30].

On the contrary, PfHRP2 repeat types obtained within this study showed certain difference from African and global reports in a number of ways. First, PfHRP2 type 12 repeat was found in a few isolates (10.5%) this did not align with previous studies from Kenya [29], Ghana [30] and Central America [35]. However, studies from Senegal [33] showed the presence of type 12 repeats in a few isolates similar to the present study. Second, this study did not find type 9 and 11 repeats isolates which is consistent with most studies from African countries [28–30], but type 11 repeats were reported in a few isolates from Madagascar [15], while type 9 was reported from Senegalese isolates [33]. Third, types 14–27 were completely absent in all isolates in this study. These results disagree with other studies, which reported the rare occurrence of type 14 from Nigeria [8] and Madagascar [15], and type 19 from Kenya [29]. The possible explanation for such varied distribution repeat types may be due to random mutation and local selection or directional spread of deleted strains of *pfhrp*2 /3 throughout the world [36].

Concerning PfHRP3, the majorities of sequences started with type 1 and ended with a type 4 repeats in the Ethiopia isolates, which agrees with previous studies [29, 31]. Moreover, all isolates of PfHRP3 amino acid sequence contained a singleton non-repeating sequence [11]. The findings of PfHRP3 repeat types in the present study are consistent with previous reports from Kenya [29], Yemen [28], Ghana [30], and globally [8]. On the other hand, type 2 repeat was absent in all isolates of PfHRP3 sequence in Ethiopia as well as other parts of Africa [28–30], but type 2 repeat has been reported in PfHRP3 in few isolates from Kenya [29] and India [31]. This variation observed in PfHRP2 and PfHRP3 repeat types might be due to differences in geographical and transmission settings [8, 37, 38], frequency of exposure of drug and level of immunity of study participants [39, 40], clinical versus asymptomatic study participants, and methods used for molecular analysis.

Interestingly, of all the novels repeat types identified in this study, 15 in PfHRP2 and 12 in PfHRP3 have not been reported elsewhere. Among all novel PfHRP2 repeat types, two unique repeat types (type 7-AHH**D**D, type 10-AHHAA**T**HHATD) and one unique repeat type (type-AHHAAAHHA**N**D) were found with low frequency in Ethiopia and also in a previous study from Ghana [30] and Kenya [29]. Among all novel PfHRP3 repeat types, a unique repeat type (type 20- SHHD**G)** was detected with high frequency in Ethiopia as well as in Kenya [29], China-Myanmar border [41], and India [31]. The emergence of novel PfHRP2 and PfHRP3 repeat types at low frequency could be initiated by replacement or deletion of one or more amino acid repeat type [42–44].

Parasite densities and genetic variation *pfhrp2/3* gene are the two most important factors that affect the performance of PfHRP2 RDT [8, 16, 21]. In this study, a high proportion of PfHRP2 RDT positive samples was observed with parasitaemia > 250 parasites/µl, but substantial numbers of PfHRP2 RDT false negative samples were detected in submicroscopic infections similar to previous reports [8, 45].

According to Baker's model, the Ethiopian isolates mostly belong to groups A and B were predicated to be detected by PfHRP2 RDT at parasitaemia level > 250 parasite/μl and thus aligns with previous studies found in Senegal [33], Madagascar [15], and India [46]. Interestingly, most of *P. falciparum* isolates in group C were detectable by PfHRP2 RDT at parasitaemia level ≥ 200 parasite/μl. This finding partially disagrees with the Baker’ model [11] that predicts if the length of repeat types 2 and 7 is below 43 (as in group C), it will alter detection sensitivity of PfHRP2 RDT and lead to false negative results [33].

In this study, the length of amino acid repeat in PfHRP2 (type 1, 2, 7) and shared repeats in PfHRP2 and PfHRP3 (type 1, 4, 7) were not statistically associated with the performance of PfHRP2 RDT, consistent with previous study [29]. Instead, PfHRP2 RDT positivity was significantly improved as the length of PfHRP3 repeat type 16 and 17 increased. Types 1, 4 and 7 were common repeat types in both PfHRP2 and PfHRP3 amino acid sequence, whereas types 16 and 17 were identified only in PfHRP3 in high frequency among all isolates. In line with this, PfHRP2 and PfHRP3 exhibit structural homology as exon 2 in both *pfhrp2* and *pfhrp3* encodes similar amino acids and cross reaction may play a role in the diagnosis of falciparum malaria [7, 12]. Moreover, novel PfHRP2 and PfHRP3 repeat variants detected in this study might influence the binding affinity to monoclonal antibody and affect the sensitivity of PfHRP2 RDTs [12]. As results, considerable proportions of false negative results were found in this study by PfHRP2 RDT, 38.5% of PfHRP2 and 37.5% of PfHRP3 sequence with novel variants.

## Limitations

This study had two limitations. First, the samples represented a limited geographical area from Assoa, Ethiopia. Second, this study did not assess cross reactivity of the possible epitopes based on the amino acid repeat sequence of PfHRP2 and PfHRP3 using specific monoclonal antibodies.

## Conclusion

The present study indicated, for the first time, the presence of extensive existence of genetic variability of PfHRP2 and PfHRP3 amino acid repeats including novel repeats in *P. falciparum* isolates within and between the study sites in Ethiopia. There is a need to closely monitor the performance of PfHRP2 RDT and examine the distribution of novel repeat type and shared repeat in PfHRP2/3, and unique repeat found in PfHRP3 broadly in Ethiopia.

## Supplementary Information

**Additional file 1.** The map showing the study area in Assosa zone. The map generated using ArcGIS version 10.0 software.

**Additional file 2.** Primer sequences, PCR conditions and expected amplicon sizes of *Pfhrp2* *exon2* and *Pfhrp3 exon2*.

**Additional file 3.** Distribution of PfHRP2 and PfHRP3 amino acid repeat by study site.

**Additional file 4.** BLASTP of Ethiopian PfHRP2 sequences for 4 Pattern and 25 distinct haplotype isolates.

**Additional file 5.** BLASTP of Ethiopian PfHRP3 sequences for eight different Pattern.

**Additional file 6.** Comparison of Ethiopia PfHRP2 novel repeat with other countries.

**Additional file 7.** Comparisons of Ethiopian PfHRP3 novel variants with others.

**Additional file 8.** Baker repeats types and P. falciparum infection diagnosis . PfHRP2 sequence isolates classified into four groups according to the frequency number of types 2 × type 7 repeats: group A (≥100, very sensitive), group B (50-99, sensitive), group C (<43, non-sensitive) and group I (44-49, borderline) as describe previously (Baker et al, 2010).

**Additional file 9.** PfHRP2 RDT and microscopy results in Pfhrp3 sequence isolates.

**Additional file 10.** Effect of the number of PfHRP2 and PfHRP3 amino acid repeat on the performance of PfHRP2 RDT.

## Data Availability

The datasets used and/or analysed during in this study are included in this article and available from the corresponding author on reasonable request.

## Abbreviations

RDT: Rapid diagnostic tests RDT
HRP2: Histidine-Rich Protein 2/3
*pfhrp*2*/3*gene: *P. falciparum* Histidine-rich protein 2 and 3 genes
DBS: Dried blood spots
PCR: Polymerase chain reaction
qPCR: Quantitative polymerase chain reaction
SPSS: Statistical Package for Social Sciences
NCBI: National Center for Biotechnology Information’s
BLASTP: Basic Local Alignment Search Tool for Protein analysis

## References

[1] WHO (2019). World malaria report 2019.

[2] Cowman AF, Healer J, Marapana D, Marsh K (2016). Malaria: biology and disease. Cell.

[3] WHO (2015). Global Technical Strategy for Malaria 2016–2030.

[4] WHO (2011). Good practices for selecting and procuring rapid diagnostic tests for malaria.

[5] Murray CK, Gasser RA, Magill AJ, Miller RS (2008). Update on rapid diagnostic testing for malaria. J Clin Microbiol.

[6] Jain P, Chakma B, Patra S, Goswami P (2014). Potential biomarkers and their applications for rapid and reliable detection of malaria. Biomed Res Int..

[7] Wellems TE, Howard RJ (1986). Homologous genes encode two distinct histidine-rich proteins in a cloned isolate of *Plasmodium falciparum*. Proc Natl Acad Sci USA.

[8] Baker J, Ho M-F, Pelecanos A, Gatton M, Chen N, Abdullah S (2010). Global sequence variation in the histidine-rich proteins 2 and 3 of *Plasmodium falciparum*: implications for the performance of malaria rapid diagnostic tests. Malar J.

[9] Sharma Y (1988). Genomic organization, structure and possible function of histidine-rich proteins of malaria parasites. Int J Biochem.

[10] Rock E, Marsh K, Saul A, Wellems T, Taylor DW, Maloy W (1987). Comparative analysis of the *Plasmodium falciparum* histidine-rich proteins HRP-I, HRP-II and HRP-III in malaria parasites of diverse origin. Parasitology.

[11] Baker J, McCarthy J, Gatton M, Kyle DE, Belizario V, Luchavez J (2005). Genetic diversity of *Plasmodium falciparum* histidine-rich protein 2 (PfHRP2) and its effect on the performance of PfHRP2-based rapid diagnostic tests. J Infect Dis.

[12] Lee N, Baker J, Andrews KT, Gatton ML, Bell D, Cheng Q (2006). Effect of sequence variation in *Plasmodium falciparum* histidine-rich protein 2 on binding of specific monoclonal antibodies: Implications for rapid diagnostic tests for malaria. J Clin Microbiol.

[13] Levinson G, Gutman G (1987). Slipped-strand mispairing: a major mechanism for DNA sequence evolution. Mol Biol Evol.

[14] Baker J, Gatton ML, Peters J, Ho M-F, McCarthy JS (2011). Transcription and expression of *Plasmodium falciparum* histidine-rich proteins in different stages and strains: implications for rapid diagnostic tests. PLoS ONE..

[15] Mariette N, Barnadas C, Bouchier C, Tichit M, Ménard D (2008). Country-wide assessment of the genetic polymorphism in *Plasmodium falciparum* and *Plasmodium vivax* antigens detected with rapid diagnostic tests for malaria. Malar J.

[16] Cheng Q, Gatton ML, Barnwell J, Chiodini P, McCarthy J, Bell D (2014). *Plasmodium falciparum* parasites lacking histidine-rich protein 2 and 3: a review and recommendations for accurate reporting. Malar J.

[17] Alemayehu GS, Blackburn K, Lopez K, Dieng CC, Lo E, Janies D (2021). Detection of high prevalence of *Plasmodium falciparum* histidine-rich protein 2/3 gene deletions in Assosa zone, Ethiopia: implication for malaria diagnosis. Malar J.

[18] Beshir KB, Sepúlveda N, Bharmal J, Robinson A, Mwanguzi J, Busula AO (2017). *Plasmodium falciparum* parasites with histidine-rich protein 2 (pfhrp2) and pfhrp3 gene deletions in two endemic regions of Kenya. Sci Rep.

[19] Hamid MA, Awad-Elgeid M, Nasr A (2017). Gene variation and suspected *Plasmodium falciparum* histidine-rich protein 2 gene deletion and its impact on sensitivity of malaria rapid diagnostic tests in Sudan. BMJ Glob Health.

[20] Amoah LE, Abankwa J, Oppong A (2016). *Plasmodium falciparum* histidine rich protein-2 diversity and the implications for PfHRP 2: based malaria rapid diagnostic tests in Ghana. Malar J.

[21] WHO (2018). Protocol for estimating the prevalence of pfhrp2/pfhrp3 gene deletions among symptomatic falciparum patients with false-negative RDT results.

[22] Daniel WW, Cross CL (2018). Biostatistics: a foundation for analysis in the health sciences.

[23] Geleta G, Ketema T (2016). Severe malaria associated with *Plasmodium falciparum* and *P. vivax* among children in Pawe Hospital, Northwest Ethiopia. Malar Res Treat..

[24] WHO (2016). Malaria microscopy quality assurance manual-version 2.

[25] Baidjoe A, Stone W, Ploemen I, Shagari S, Grignard L, Osoti V (2013). Combined DNA extraction and antibody elution from filter papers for the assessment of malaria transmission intensity in epidemiological studies. Malar J.

[26] Rougemont M, Van Saanen M, Sahli R, Hinrikson HP, Bille J, Jaton K (2004). Detection of four *Plasmodium* species in blood from humans by 18S rRNA gene subunit-based and species-specific real-time PCR assays. J Clin Microbiol.

[27] Parr JB, Anderson O, Juliano JJ, Meshnick SR (2018). Streamlined, PCR-based testing for pfhrp2-and pfhrp3-negative *Plasmodium falciparum*. Malar J.

[28] Atroosh WM, Al-Mekhlafi HM, Al-Jasari A, Sady H, Al-Delaimy AK, Nasr NA (2015). Genetic variation of pfhrp2 in *Plasmodium falciparum* isolates from Yemen and the performance of HRP2-based malaria rapid diagnostic test. Parasit Vectors.

[29] Nderu D, Kimani F, Thiong’o K, Karanja E, Akinyi M, Too E (2019). *Plasmodium falciparum* histidine-rich protein (PfHRP2 and 3) diversity in Western and Coastal Kenya. Sci Rep..

[30] Addai-Mensah O, Dinko B, Noagbe M, Ameke SL, Annani-Akollor ME, Owiredu E-W (2020). *Plasmodium falciparum* histidine-rich protein 2 diversity in Ghana. Malar J.

[31] Bharti PK, Singh Chandel H, Krishna S, Nema S, Ahmad A (2017). Sequence variation in *Plasmodium falciparum* histidine rich proteins 2 and 3 in Indian isolates: implications for malaria rapid diagnostic test performance. Sci Rep.

[32] Deme AB, Park DJ, Bei AK, Sarr O, Badiane AS, Gueye PE (2014). Analysis of pfhrp2 genetic diversity in Senegal and implications for use of rapid diagnostic tests. Malar J.

[33] Wurtz N, Fall B, Bui K, Pascual A, Fall M, Camara C (2013). Pfhrp2 and pfhrp3 polymorphisms in *Plasmodium falciparum* isolates from Dakar, Senegal: impact on rapid malaria diagnostic tests. Malar J.

[34] Abdallah JF, Okoth SA, Fontecha GA, Torres REM, Banegas EI, Matute ML (2015). Prevalence of *pfhrp*2 and *pfhrp*3 gene deletions in Puerto Lempira. Honduras Malar J.

[35] Fontecha G, Pinto A, Escobar D, Matamoros G, Ortiz B (2019). Genetic variability of *Plasmodium falciparum* histidine-rich proteins 2 and 3 in Central America. Malar J.

[36] Baker J, Gatton ML, Peters J, Ho M-F, McCarthy JS, Cheng Q (2011). Transcription and expression of *Plasmodium falciparum* histidine-rich proteins in different stages and strains: implications for rapid diagnostic tests. PLoS ONE..

[37] Watson OJ, Verity R, Ghani AC, Garske T, Cunningham J, Tshefu A (2019). Impact of seasonal variations in *Plasmodium falciparum* malaria transmission on the surveillance of pfhrp2 gene deletions. Elife..

[38] Pringle JC, Wesolowski A, Berube S, Kobayashi T, Gebhardt ME, Mulenga M (2019). High *Plasmodium falciparum* genetic diversity and temporal stability despite control efforts in high transmission settings along the international border between Zambia and the Democratic Republic of the Congo. Malar J.

[39] Markwalter CF, Mudenda L, Leelawong M, Kimmel DW, Nourani A, Mbambara S (2018). Evidence for histidine-rich protein 2 immune complex formation in symptomatic patients in Southern Zambia. Malar J.

[40] Apinjoh TO, Ouattara A, Titanji VP, Djimde A, Amambua-Ngwa A (2019). Genetic diversity and drug resistance surveillance of *Plasmodium falciparum* for malaria elimination: is there an ideal tool for resource-limited sub-Saharan Africa?. Malar J.

[41] Li P, Xing H, Zhao Z, Yang Z, Cao Y, Li W (2015). Genetic diversity of *Plasmodium falciparum* histidine-rich protein 2 in the China-Myanmar border area. Acta Trop.

[42] Akinyi S, Hayden T, Gamboa D, Torres K, Bendezu J, Abdallah JF (2013). Multiple genetic origins of histidine-rich protein 2 gene deletion in *Plasmodium falciparum* parasites from Peru. Sci Rep.

[43] Kemp D, Thompson J, Walliker D, Corcoran L (1987). Molecular karyotype of *Plasmodium falciparum*: conserved linkage groups and expendable histidine-rich protein genes. Proc Natl Acad Sci USA.

[44] Gamboa D, Ho M-F, Bendezu J, Torres K, Chiodini PL, Barnwell JW (2010). A large proportion of *P. falciparum* isolates in the Amazon region of Peru lack pfhrp2 and pfhrp3: implications for malaria rapid diagnostic tests. PLoS ONE..

[45] WHO (2017). Malaria rapid diagnostic test performance: results of WHO product testing of malaria RDTs: round 7 (2015–2016).

[46] Kumar N, Singh JP, Pande V, Mishra N, Srivastava B, Kapoor R (2012). Genetic variation in histidine rich proteins among Indian *Plasmodium falciparum* population: possible cause of variable sensitivity of malaria rapid diagnostic tests. Malar J.

