# Supplementary material for: Genetic variation of Plasmodium falciparum histidine-rich protein 2 and 3 in Assosa zone, Ethiopia: its impact on the performance of malaria rapid diagnostic tests
Source: Malar J. 2021 Oct 9;20:394. doi: 10.1186/s12936-021-03928-3 (PMC8502267; doi:10.1186/s12936-021-03928-3)
Supplement: Supplementary file 1 — Additional file 1. The map showing the study area in Assosa zone. The map generated using ArcGIS version 10.0 software. [file 12936_2021_3928_MOESM1_ESM.docx]

1. Additional file 1. The map showing the study area in Assosa zone. The map generated using ArcGIS version 10.0 software.
