# Supplementary material for: Genetic variation of Plasmodium falciparum histidine-rich protein 2 and 3 in Assosa zone, Ethiopia: its impact on the performance of malaria rapid diagnostic tests
Source: Malar J. 2021 Oct 9;20:394. doi: 10.1186/s12936-021-03928-3 (PMC8502267; doi:10.1186/s12936-021-03928-3)
Supplement: Supplementary file 2 — Additional file 2. Primer sequences, PCR conditions and expected amplicon sizes of Pfhrp2 exon2 and Pfhrp3 exon2. [file 12936_2021_3928_MOESM2_ESM.docx]

| Additional file 2 . Primer sequences, PCR conditions and expected amplicon sizes of *Pfhrp2 exon2* and *Pfhrp3 exon2* | | | | | |
| --- | --- | --- | --- | --- | --- |
| **Gene** | **Primer** | **Primer sequences (5′→3′)** | **PCR Condition** | **Amplicon size** | **Reference** |
| PF3D7_0831800 | Pfhrp2_ex2_F | ATT CCG CAT TTA ATA ATAACT TGT GTAGC | 95 °C × 15 min;40 cycles of 94 °C × 1 min, | 600-960bp | Parr et al 2018 |
| (Pfhrp2 exon2) | Pfhrp2_ex2_R | ATG GCG TAG GCA ATGTGT GG | 59 °C × 1 min,72 °C × 1 min;72 °C × 10 min |  |  |
| PF3D7_1372200 | Pfhrp3_ex2_F | AAT GCA AAA GGA CTT AAT TC |  | 600-650bp | Parr et al 2018 |
|  |  |  | 95 °C × 15 min; 40 cycles of 94 °C × 1 min, |  |  |
| (Pfhrp3 exon 2) | Pfhrp3_ex2_R | TGG TGT AAG TGA TGC GTA GT | 55 °C × 1 min, 60 °C × 1 min; 60 °C × 10 min |  |  |
