# Supplementary material for: Genetic variation of Plasmodium falciparum histidine-rich protein 2 and 3 in Assosa zone, Ethiopia: its impact on the performance of malaria rapid diagnostic tests
Source: Malar J. 2021 Oct 9;20:394. doi: 10.1186/s12936-021-03928-3 (PMC8502267; doi:10.1186/s12936-021-03928-3)
Supplement: Supplementary file 3 — Additional file 3. Distribution of PfHRP2 and PfHRP3 amino acid repeat by study site. [file 12936_2021_3928_MOESM3_ESM.docx]

| **Additional File 3 : Distribution of PfHRP2 and PfHRP3 amino acid repeat by study site** | | | | | | | | |
| --- | --- | --- | --- | --- | --- | --- | --- | --- |
| Type of repeat | Repeat frequency of PfHRP2 in the Study sites | | | | Repeat frequency of PfHRP3 in the Study sites | | | |
|  | Sherkole  (N=24) | Bambasi  (N=15) | Kurmuk  (N=7) | Assosa  (N=2) | Sherkole  (N=34) | Bambasi  (N=32) | Kurmuk  (N=20) | Assosa  (N=2) |
|  | n (%) | n(%) | n(%) | n(%) | n(%) | n(%) | n(%) | n(%) |
| 1 | 24(100) | 15(100) | 7(100) | 2(100) | 32(94.1) | 30(93.8) | 20(100) | 1(50) |
| 2 | 24(100) | 15(100) | 7(100) | 2(100) | 0(0.0) | 0(0.0) | 0(0.0) | 0(0.0) |
| 3 | 20(83.3) | 14(93.3) | 5(71.4) | 2(100) | 0(0.0) | 0(0.0) | 0(0.0) | 0(0.0) |
| 4 | 15(62.5) | 3(20.0) | 1(14.3) | 1(50) | 29(85.3) | 27(84.4) | 20(100) | 2(100) |
| 5 | 19(79.2) | 10(66.7) | 6(85.7) | 1(50) | 0(0.0) | 0(0.0) | 0(0.0) | 0(0.0) |
| 6 | 23(95.8) | 15(100) | 6(85.7) | 2(100) | 0(0.0) | 0(0.0) | 0(0.0) | 0(0.0) |
| 7 | 24(100) | 15(100) | 7(100) | 2(100) | 33(97.1) | 31(96.9) | 20(100) | 2(100) |
| 8 | 20(83.3) | 15(100) | 7(100) | 2(100) | 0(0.0) | 0(0.0) | 0(0.0) | 0(0.0) |
| 9 | 0(0.0) | 0(0.0) | 0(0.0) | 0(0.0) | 0(0.0) | 0(0.0) | 0(0.0) | 0(0.0) |
| 10 | 23(95.8) | 14(93.3) | 7(100) | 2(100) | 0(0.0) | 0(0.0) | 0(0.0) | 0(0.0) |
| 11 | 0(0.0) | 0(0.0) | 0(0.0) | 0(0.0) | 0(0.0) | 0(0.0) | 0(0.0) | 0(0.0) |
| 12 | 3(12.5) | 1(6.7) | 1(14.3) | 0(0.0) | 0(0.0) | 0(0.0) | 0(0.0) | 0(0.0) |
| 13 | 3(12.5) | 2(13.3) | 0(0.0) | 0(0.0) | 0(0.0) | 0(0.0) | 0(0.0) | 0(0.0) |
| 14 | 0(0.0) | 0(0.0) | 0(0.0) | 0(0.0) | 0(0.0) | 0(0.0) | 0(0.0) | 0(0.0) |
| 15 | 0(0.0) | 0(0.0) | 0(0.0) | 0(0.0) | 33(97.1) | 30(93.8) | 20(100) | 1(50) |
| 16 | 0(0.0) | 0(0.0) | 0(0.0) | 0(0.0) | 34(100) | 32(100) | 20(100) | 2(100) |
| 17 | 0(0.0) | 0(0.0) | 0(0.0) | 0(0.0) | 34(100) | 32(100) | 20(100) | 2(100) |
| 18 | 0(0.0) | 0(0.0) | 0(0.0) | 0(0.0) | 34(100) | 32(100) | 19(95.0) | 2(100) |
| 19 | 0(0.0) | 0(0.0) | 0(0.0) | 0(0.0) | 0(0.0) | 0(0.0) | 0(0.0) | 0(0.0) |
| 20 | 0(0.0) | 0(0.0) | 0(0.0) | 0(0.0) | 34(100) | 32(100) | 19(95.0) | 2(100) |
| 21 | 0(0.0) | 0(0.0) | 0(0.0) | 0(0.0) | 0(0.0) | 0(0.0) | 0(0.0) | 0(0.0) |
| 22 | 0(0.0) | 0(0.0) | 0(0.0) | 0(0.0) | 0(0.0) | 0(0.0) | 0(0.0) | 0(0.0) |
| 23 | 0(0.0) | 0(0.0) | 0(0.0) | 0(0.0) | 0(0.0) | 0(0.0) | 0(0.0) | 0(0.0) |
| 24 | 0(0.0) | 0(0.0) | 0(0.0) | 0(0.0) | 0(0.0) | 0(0.0) | 0(0.0) | 0(0.0) |
| N=Total number of sequences analyzed in the study sites, %=Percentage of samples in each repeat type. | | | | | | | | |

N=Repeat frequency in the Shr/Bamb/Kur/Ass isolates
