# Supplementary material for: Genetic variation of Plasmodium falciparum histidine-rich protein 2 and 3 in Assosa zone, Ethiopia: its impact on the performance of malaria rapid diagnostic tests
Source: Malar J. 2021 Oct 9;20:394. doi: 10.1186/s12936-021-03928-3 (PMC8502267; doi:10.1186/s12936-021-03928-3)
Supplement: Supplementary file 4 — Additional file 4. BLASTP of Ethiopian PfHRP2 sequences for 4 Pattern and 25 distinct haplotype isolates. [file 12936_2021_3928_MOESM4_ESM.docx]

| Additional File 4. BLASTP of Ethiopian PfHRP2 sequences for 4 Pattern and 25 distinct haplotype isolates | | | | | |
| --- | --- | --- | --- | --- | --- |
| **Type of Pattern /Sample ID** | **% identity** | | **E-value** | **Country** | **Accession number⁕** |
| Pattern I(HKum47, LBab57) | 88.50% | | 8.00E-89 | Kenya | QBC65676.1 |
| Pattern II (Hbab 100, HBab64,66,68, 75, 80, 81, Hkum35) | 94.92% | | 5.00E-89 | Papua new Guinea | AUB13314.1 |
| Pattern III (LShr 108, LShr 73, LShr 133) | 97.01% | | 5.00E-84 | Kenya | QBC65625.1 |
| Pattern V (HShr118, Lkum 74) | 92.76% | | 2.00E-71 | Madagascar | ACE88446.1 |
| LShr5 | 95.38% | | 4.00E-88 | Kenya | QBC65714.1 |
| HShr161 | 100% | | 2.00E-95 | Kenya | ACZ25641.1 |
| HShr17 | 98.90% | | 2.00E-106 | kenya | QBC65631.1 |
| HShr14 | 95.20% | | 2.00E-94 | Myanmar | ARQ79822.1 |
| HAss42 | 96.88% | | 4.00E-92 | Kenya | QBC65669.1 |
| HShr122 | 97.25% | | 1.00E-92 | Kenya | QBC65714.1 |
| HShr80 | 94.98% | | 9.00E-89 | Unkown | QKY74468.1 |
| HBab37 | 100% | | 2.00E-93 | Kenya | QBC65627 |
| Hkum11 | 97.27% | | 7.00E-94 | Kenya | QBC65714.1 |
| HShr146 | 98.82% | | 1.00E-96 | Madagascar | ACE88431.1 |
| Hkum67 | 92.40% | | 3.00E-75 | Kenya | QBC65740.1 |
| HShr148 | 98.37% | | 7.00E-92 | Kenya | QBC65688.1 |
| HShr44 | 92.71% | | 1.00E-80 | Kenya | QBC65514.1 |
| LBab98 | 93.37% | 1.00E-71 | | India | AQY61980.1 |
| Lkum17 | 93.50% | 3.00E-76 | | India | AQY61916.1 |
| HBab118 | 97.50% | 4.00E-85 | | Madagascar | ACE88453.1 |
| HShr45 | 94.98% | 1.00E-80 | | Kenya | QBC65526.1 |
| LShr 171 | 96.97% | 2.00E-84 | | kenya | QBC65691.1 |
| HAss39 | 92.75% | 1.00E-94 | | kenya | QBC65513.1 |
| HShr70 | 94.54% | | 3.00E-80 | Kenya | QBC65629.1 |
| HBab18 | 94.39% | | 1.00E-79 | ?? | QKY74469.1 |
| Lkum28 | 98.37% | | 2.00E-64 | Kenya | ACZ25641.1 |
| LShr44 | 95.72% | | 1.00E-62 | ?? | QKY74481.1 |
| HShr105 | 100.00% | | 1.00E-05 | ?? | CAA49544.1 |
| HShr126 | 100.00% | | 3.00E-06 | ?? | QKY4500.1 |
| ⁕Accession numbers with the highest percentage identity selected among BLASTP hits. | | | | | |
