# Supplementary material for: Genetic variation of Plasmodium falciparum histidine-rich protein 2 and 3 in Assosa zone, Ethiopia: its impact on the performance of malaria rapid diagnostic tests
Source: Malar J. 2021 Oct 9;20:394. doi: 10.1186/s12936-021-03928-3 (PMC8502267; doi:10.1186/s12936-021-03928-3)
Supplement: Supplementary file 5 — Additional file 5. BLASTP of Ethiopian PfHRP3 sequences for eight different Pattern. [file 12936_2021_3928_MOESM5_ESM.docx]

| Additional file 5 . BLASTP of Ethiopian PfHRP3 sequences for eight different Pattern | | | | |
| --- | --- | --- | --- | --- |
| **Type of Pattern /No of Isolates** | **% identity** | **E-value** | **Country** | **Accession number⁕** |
| Pattern I ( 71 isolates) | 100% | **1.00E-93** | Kenya | QBC65798.1 |
| Pattern II ( one isolate) | 99.35% | **2.00E-66** | Kenya | QBC65844.1 |
| Pattern III (one isolate) | 97.30% | **3.00E-22** | Kenya | QBC65842.1 |
| Pattern IV( two isolates) | 93.55% | **6.00E-29** | Kenya | QBC65842.1 |
| Pattern V ( 9 isolates) | 99.50% | **5.00E-100** | Kenya | QBC65801.1 |
| Pattern VI ( One isolates) | 99.26% | **4.00E-62** | India | AQY62129.1 |
| Pattern VII ( two isolates) | 100% | **1.00E-72** | Kenya | QBC65846.1 |
| Pattern VIII ( One isolates) | 96.47% | **3.00E-28** | Kenya | QBC65842.1 |
| ⁕Accession numbers with the highest percentage identity selected among BLASTP hits | | | | |
