# Supplementary material for: Genetic variation of Plasmodium falciparum histidine-rich protein 2 and 3 in Assosa zone, Ethiopia: its impact on the performance of malaria rapid diagnostic tests
Source: Malar J. 2021 Oct 9;20:394. doi: 10.1186/s12936-021-03928-3 (PMC8502267; doi:10.1186/s12936-021-03928-3)
Supplement: Supplementary file 6 — Additional file 6. Comparison of Ethiopia PfHRP2 novel repeat with other countries. [file 12936_2021_3928_MOESM6_ESM.docx]

Additional file 6: Comparison of Ethiopia PfHRP2 novel repeat with other countries

|  | | | |  | | | | Novel repeat | | | |  | | | |  | | | | | |  | | | | | | |  | | |  | |  |  |  |
| --- | --- | --- | --- | --- | --- | --- | --- | --- | --- | --- | --- | --- | --- | --- | --- | --- | --- | --- | --- | --- | --- | --- | --- | --- | --- | --- | --- | --- | --- | --- | --- | --- | --- | --- | --- | --- |
| Type of repeat | | | | Known amino acid repeat | | | | Ethiopian | | | | Kenya | | | | Ghana | | | | | | India | | | | | | | Myanmar | | China-Myanmar border | | |  |  |  |
|  | | | |  | | | | PfHRP2 | | | | PfHRP2 | | | | PfHRP2 | | | | | | PfHRP2 | | | | | | | PfHRP2 | | PfHRP2 | | |  |  |  |
| 1 | | | | AHHAHHVAD | | | | - | | | | AHHAHHVA**Y** | | | | AHHAHHVA**Y** | | | |  | | | | | | AHHAHHVA**Y** | | | | | | | AHHAHHVA**Y** | | |  |
|  |  |  |  |  |  |  |  |  |  |  |  | AHHAHHV**P**D | | | | AHH**T**HHVAD | | | |  | | | | | | AH**R**AHHVAD | | | | | | |  | | |  |
|  |  |  |  |  |  |  |  |  |  |  |  | AHH**T**HHVAD | | | | A**PD**AHHVAD | | | |  | | | | | | AHHA**R**HVAD | | | | | | |  | | |  |
|  | | | |  | | | |  | | | |  | | | |  | | | |  | | | | | | AH**P**AHHVAD | | | | | | |  | | |  |
|  | | | |  | | | |  | | | |  | | | |  | | | |  | | | | | | AHHAHH**E**AD | | | | | | |  | | |  |
|  | | | |  | | | |  | | | |  | | | |  | | | |  | | | | | | A**R**HAHHVAD | | | | | | |  | | |  |
| 2 | | | | AHHAHHAAD | | | | AHHAHH**E**AD | | | | AHHA**D**HAAD | | | | AHHAHHA**P**D | | | | AHH**S**HHAAD | | | | | | **V**HHAHHAAD | | | | | | | AHHA**Y**HAAD | | | |
|  |  |  |  |  |  |  |  | AHHA**Q**HAAD | | | | AHHAHHA**D**D | | | | AHHAHHA**D**D | | | |  | | | | | | AHHAHHAA**G** | | | | | | | AHHAH**Y**AAD | | | |
|  |  |  |  |  |  |  |  | AHHAHHA**HH** | | | | AHHAHHAA**H** | | | | AHHAHHAD**H** | | | | | |  | | | | | AHHAHH**T**AD | | | | | | A**Y**HAHHAAD | | | |
|  |  |  |  |  |  |  |  | A**PD**HHAHH | | | | AHHAHHA**DH** | | | |  | | | | | |  | | | | | AHHA**R**HAAD | | | | | | AHHAH**R**AAD | | | |
|  |  |  |  |  |  |  |  | AHHAH**V**AAD | | | | AHHAHHA**P**D | | | |  | | | | | |  | | | | | AHH**T**HHAAD | | | | | | AHHA**R**HAAD | | | |
|  |  |  |  |  |  |  |  | AHHAHHAA**Y** | | | | AHHAHHA**PH** | | | |  | | | | | |  | | | | | A**R**HAHHAAD | | | | | | AHHAHH**T**AD | | | |
|  | | | |  | | | |  | | | |  | | | |  | | | | | |  | | | | | | |  | | | | AHHAH**T**AAD | | | |
|  | | | |  | | | |  | | | |  | | | |  | | | | | |  | | | | | | |  | | | | AHHAHHA**V**D | | |  |
|  | | | |  | | | |  | | | |  | | | |  | | | | | |  | | | | | | |  | | | | AHHAHHAA**G** | | |  |
| 3 | | | | AHHAHHAAY | | | | AHHAH**Y**AAY | | | |  | | | |  | | | | | |  | | | | | | |  | | | |  | | |  |
| 4 | | | | AHH | | | |  | | | | AH**Q** | | | |  | | | | | |  | | | | | | |  | | | | **T**HH | | |  |
|  | | | |  | | | |  | | | | A**D**H | | | |  | | | | | |  | | | | | | |  | | | | A**R**H | | |  |
|  | | | |  | | | |  | | | |  | | | |  | | | | | |  | | | | | | |  | | | | AH**R** | | |  |
|  | | | |  | | | |  | | | |  | | | |  | | | | | |  | | | | | | |  | | | | A**Q**H | | |  |
|  | | | |  | | | |  | | | |  | | | |  | | | | | |  | | | | | | |  | | | | AH**Y** | | |  |
| 5 | | | | AHHAHHASD | | | | | | | | AHHA**P**HASD | | | | AHHA**P**HASD | | | | | | AHHAHH**V**SD | | | | | | |  | | | | AHHA**Q**HASD | | |  |
|  | | | |  | | | |  | | | | AHH**D**HHASD | | | |  | | | | | |  | | | | | | |  | | | |  | | |  |
| 6 | | | | AHHATD | | | |  | | | | AHHAT**H** | | | |  | | | | | |  | | | | | | | **V**HHATD | | | | AH**L**ATD | | |  |
|  | | | |  | | | |  | | | |  | | | |  | | | | | |  | | | | | | | AHHA**I**D | | | |  | | |  |
|  | | | |  | | | |  | | | |  | | | |  | | | | | |  | | | | | | | **D**HHATD | | | |  | | |  |
|  | | | |  | | | |  | | | |  | | | |  | | | | | |  | | | | | | | AHHA**P**D | | | |  | | |  |
| 7 | | | | AHHAAD | | | | AHHA**VH** | | | | AHHA**P**D | | | | AHHAA**H** | | | | | | AHH**V**AD | | | | | | | **†**HHAAD | | | | A**R**HAAD | | |  |
|  |  |  |  |  |  |  |  | AHHA**D**D | | | | AHHAA**H** | | | | AHHA**D**D | | | | | |  | | | | | | | AHHAA**A** | | | | AH**R**AAD | | |  |
|  |  |  |  |  |  |  |  | AHHA**V**D | | | | AHHA**D**D | | | |  | | | | | |  | | | | | | |  | | | | AHHA**P**D | | |  |
|  | | | |  | | | |  | | | | AHHA**H**D | | | |  | | | | | |  | | | | | | |  | | | | AHHAA**E** | | |  |
|  | | | |  | | | |  | | | | AHHA**N**D | | | |  | | | | | |  | | | | | | |  | | | |  | | |  |
|  | | | |  | | | |  | | | | AHHA**NH** | | | |  | | | | | |  | | | | | | |  | | | |  | | |  |
| 8 | | | | AHHAAY | | | |  | | | | AHHA**D**Y | | | |  | | | | | | AHHA**S**Y | | | | | | |  | | | |  | | |  |
| 10 | | | | AHHAAAHHATD | | | | AHHAAAHHA**†** | | | | AHHAAAH**D**A**N**D | | | |  | | | | | |  | | | | | | |  | | | | AH**Y**AAAHHATD | | |  |
|  |  |  |  |  |  |  |  | AHHAAA**D**HHATD | | | | AHHAAAHHA**N**D | | | |  | | | | | |  | | | | | | |  | | | |  | | |  |
|  |  |  |  |  |  |  |  | AHHAAAH**Q**ATD | | | | AHHAAAHHAT**G** | | | |  | | | | | |  | | | | | | |  | | | |  | | |  |
|  |  |  |  |  |  |  |  | AHHAAAHHA**N**D | | | | AHHAA**T**HHATD | | | | AHHAA**T**HHATD | | | | | |  | | | | | | |  | | | |  | | |  |
|  |  |  |  |  |  |  |  | AHHAAAHHA**R**D | | | |  | | | |  | | | | | |  | | | | | | |  | | | |  | | |  |
|  |  |  |  |  |  |  |  | AHHAA**D**AHHATD | | | |  | | | |  | | | | | |  | | | | | | |  | | | |  | | |  |
|  |  |  |  |  |  |  |  | AHHAA**T**HHATD | | | |  | | | |  | | | | | |  | | | | | | |  | | | |  | | |  |
| 12 | | | | AHHAAAHHEAATH | | | | **V**HHAAAHHEA**PLC** | | | | A**D**HAAAH**DD**AATH | | | | AHHAAAHHEAA**S**H | | | | | |  | | | | | | |  | | | | AHHAAAH**R**EAATH | | |  |
|  | | | |  | | | |  | | | | A**D**HAAAHHEAATH | | | | AHHAAAH**D**EAA**LI** | | | | | |  | | | | | | |  | | | | AHHAAAHH**G**AATH | | |  |
|  | | | |  | | | |  | | | | AHHAAAH**DDH**ATH | | | | | | | | | |  | | | | | | |  | | | | AHHAAAHHEAAT**Q** | | |  |
|  | | | |  | | | |  | | | | AHHAAAH**D**EAATH | | | | | | | | | |  | | | | | | |  | | | |  | | |  |
|  | | | |  | | | |  | | | | AHHAAAHHEAA**A**H | | | | | | | | | |  | | | | | | |  | | | |  | | |  |
|  | | | |  | | | |  | | | | AHHAAAHHEAA**S**H | | | | | | | | | |  | | | | | | |  | | | |  | | |  |
|  | | | |  | | | |  | | | | AHHAAAHHE**S**ATH | | | | | | | | | |  | | | | | | |  | | | |  | | |  |
|  | | | |  | | | |  | | | | AHHAAAHH**H**AATH | | | | | | | | | |  | | | | | | |  | | | |  | | |  |
|  | | | |  | | | |  | | | | AHHAAAH**P**EAATH | | | | | | | | | |  | | | | | | |  | | | |  | | |  |
|  | | | |  | | | |  | | | | AHHAA**P**HHEAATH | | | | | | | | | |  | | | | | | |  | | | |  | | |  |
|  | | | |  | | | |  | | | | AHHA**D**AHH**D**AATH | | | | | | | | | |  | | | | | | |  | | | |  | | |  |
| 13 | | | | AHHASD | | | |  | | | | AHHAS**H** | | | |  | | | | | |  | | | | | | |  | | | |  | | |  |
| 20 | | | | SHHDD | | | |  | | | |  | | | |  | | | | | | SHHD**G** | | | | | | |  | |  | | |  |  |  |
| The frequency of each novel variant is low, range from 1-5.  **Note:** bold underline letter with yellow color indicate position of novel repeat with replacement of one or more amino acid compared to known repeat.**†=** indicate the position of deletion of one or more amino acid | | | | | | | | | | | | | | | | | | | | | | | | | | | | | | | | | |  |  |  |
