# Supplementary material for: Genetic variation of Plasmodium falciparum histidine-rich protein 2 and 3 in Assosa zone, Ethiopia: its impact on the performance of malaria rapid diagnostic tests
Source: Malar J. 2021 Oct 9;20:394. doi: 10.1186/s12936-021-03928-3 (PMC8502267; doi:10.1186/s12936-021-03928-3)
Supplement: Supplementary file 7 — Additional file 7. Comparisons of Ethiopian PfHRP3 novel variants with others. [file 12936_2021_3928_MOESM7_ESM.docx]

|  |  |  | Novel repeat |  |
| --- | --- | --- | --- | --- |
| Type of repeat | Known amino acid repeat | Ethiopian | Kenya | Myanmar |
|  |  | PfHRP3 | PfHRP3 | PfHRP3 |
| 1 | AHHAHHVAD | **D**HHAHHVAD | AHHAHH**G**A**E** |  |
|  |  | **P**HHAHHVAD | AHH**S**HHVAD |  |
|  |  | **SP**HAHHVAD | AH**Q**AHHVAD |  |
|  |  |  | A**Q**HAHHVAD |  |
| 4 | AHH | **V**HH |  |  |
| 7 | AHHAAD |  | AHHA**D**D |  |
|  |  |  |  |  |
| 15 | AHHAHHAAN | AHHAHHA**D**N | AHHAHHA**PH** |  |
|  |  | AHHAHH**V**AN |  |  |
| 16 | AHHAAN | AHHA**GD** | AHHAA**H** | AHHA**S**N |
|  |  | A**LL**AAN | AHHA**D**N |  |
|  |  | **S**HHAAN | AHHA**PH** |  |
|  |  |  | AHHA**S**N |  |
|  |  |  | AHH**T**AN |  |
|  |  |  | AH**Q**A**D**N |  |
|  |  |  | A**Y**HA**SH** |  |
| 17 | AHHDG |  | AHHD**E** |  |
|  |  |  | AHHD**H** |  |
|  |  |  | AH**Y**DG |  |
|  |  |  | **P**HHDG |  |
|  |  |  | **P**H**Q**DG |  |
|  |  |  | **S**HHDG |  |
| 18 | AHHDD | **E**HHD**E** | A**P**HDD |  |
|  |  | AHHD**E** |  |  |
|  |  | A**R**HDD |  |  |
| 20 | SHHDD | SHHD**G** |  | S**Y**HDD |
| The frequency of each novel variant is low, range from 1-5, **Note:** bold underline letter with yellow color indicate position of novel repeat with replacement of one or more amino acid compared to known repeat. | | | | |

Additional file 7 : Comparisons of Ethiopian PfHRP3 novel variants with others
