# Supplementary material for: Genetic variation of Plasmodium falciparum histidine-rich protein 2 and 3 in Assosa zone, Ethiopia: its impact on the performance of malaria rapid diagnostic tests
Source: Malar J. 2021 Oct 9;20:394. doi: 10.1186/s12936-021-03928-3 (PMC8502267; doi:10.1186/s12936-021-03928-3)
Supplement: Supplementary file 8 — Additional file 8. Baker repeats types and P. falciparum infection diagnosis . PfHRP2 sequence isolates classified into four groups according to the frequency number of types 2 × type 7 repeats: group A (≥100, very sensitive), group B (50-99, sensitive), group C (<43, non-sensitive) and group I (44-49, borderline) as describe previously (Baker et al, 2010). [file 12936_2021_3928_MOESM8_ESM.docx]

| Additional file 8: Baker repeats types and *P. falciparum* infection diagnosis . PfHRP2 sequence isolates classified into four groups according to the frequency number of types 2 × type 7 repeats: group A (≥100, very sensitive), group B (50-99, sensitive), group C (<43, non-sensitive) and group I (44-49, borderline) as describe previously (Baker et al, 2010) | | | | | | | |
| --- | --- | --- | --- | --- | --- | --- | --- |
| **Sample ID** | **Repeat type no** | **Group** | **Microscopy** | **PfHRP2 RDT Results** | | **Isolates with Novel PfHRP2 Repeat†** |  |
|  |  |  | **Parasite/µl** | | |  | |
| HShr14 | 98 | B | 9600 | | Positive | **Present** | |
| HShr17 | 72 | B | 240 | | Positive | Absent | |
| HShr44 | 55 | B | Neg | | Neg | **Present** | |
| HShr45 | 91 | B | 13200 | | Positive | **Present** | |
| HShr70 | 60 | B | 7200 | | Positive | **Present** | |
| HShr80 | 78 | B | 320 | | Positive | Absent | |
| HShr88 | 84 | B | 4040 | | Positive | Absent | |
| HShr96 | 84 | B | Neg | | Neg | Absent | |
| HShr105 | 20 | C | Neg | | Neg | **Present** | |
| HShr118 | 33 | C | 11880 | | Positive | Absent | |
| HShr122 | 88 | B | 160 | | Positive | Absent | |
| HShr126 | 5 | C | Neg | | Neg | Absent | |
| HShr146 | 78 | B | 8600 | | Positive | Absent | |
| HShr148 | 91 | B | 9800 | | Positive | **Present** | |
| HShr161 | 75 | B | 8760 | | Positive | Absent | |
| HBab18 | 24 | C | Neg | | Neg | **Present** | |
| HBab21 | 24 | C | 800 | | Positive | Absent | |
| HBab37 | 96 | B | Neg | | Neg | Absent | |
| HBab64 | 108 | A | 3560 | | Positive | Absent | |
| HBab66 | 108 | A | 3240 | | Positive | Absent | |
| HBab68 | 108 | A | 2880 | | Positive | Absent | |
| HBab75 | 108 | A | 3480 | | Positive | Absent | |
| HBab79 | 70 | B | Neg | | Neg | Absent | |
| HBab80 | 108 | A | 1480 | | Positive | Absent | |
| HBab81 | 108 | A | 2680 | | Positive | Absent | |
| HBab100 | 108 | A | Neg | | Neg | **Present** | |
| HBab118 | 91 | B | 400 | | Positive | Absent | |
| HKum11 | 84 | B | 160 | | Positive | Absent | |
| HKum34 | 100 | A | Neg | | Neg | Absent | |
| HKum47 | 156 | A | 8280 | | Positive | **Present** | |
| HKum67 | 100 | A | Neg | | Neg | Absent | |
| HAss39 | 78 | B | Neg | | Neg | Absent | |
| HAss42 | 84 | B | 400 | | Positive | **Present** | |
| LShr5 | 91 | B | 80 | | Positive | Absent | |
| LShr44 | 40 | C | 8000 | | Positive | **Present** | |
| LShr64 | 40 | C | 400 | | Positive | Absent | |
| LShr73 | 72 | B | Neg | | Neg | Absent | |
| LShr108 | 72 | B | 8400 | | Positive | Absent | |
| LShr116 | 40 | C | 560 | | Positive | Absent | |
| LShr133 | 78 | B | 200 | | Positive | **Present** | |
| LShr165 | 35 | C | 200 | | Positive | Absent | |
| LShr171 | 55 | B | 6000 | | Positive | Absent | |
| LBas37 | 91 | B | 3200 | | Positive | Absent | |
| LBas57 | 156 | A | 3600 | | Positive | Absent | |
| LBas98 | 52 | B | 1400 | | Positive | Absent | |
| LKum17 | 98 | B | **Neg** | | Neg | **Present** | |
| LKum28 | 48 | I | 520 | | Positive | Absent | |
| LKum74 | 33 | C | 400 | | Positive | Absent | |

† (**Present** in bold Yellow highlight) =indicate the presence of one or more novel PfHRP2 repeat type in the respective isolates
