# Supplementary material for: Genetic variation of Plasmodium falciparum histidine-rich protein 2 and 3 in Assosa zone, Ethiopia: its impact on the performance of malaria rapid diagnostic tests
Source: Malar J. 2021 Oct 9;20:394. doi: 10.1186/s12936-021-03928-3 (PMC8502267; doi:10.1186/s12936-021-03928-3)
Supplement: Supplementary file 9 — Additional file 9. PfHRP2 RDT and microscopy results in Pfhrp3 sequence isolates. [file 12936_2021_3928_MOESM9_ESM.docx]

| Additional file 9 : PfHRP2 RDT and microscopy results in Pfhrp3 sequence isolates | | | | | | | |  |
| --- | --- | --- | --- | --- | --- | --- | --- | --- |
| ID | PfHRP2RDT | | | Microscopy/Parasite/µl  **isolates with Novel PfHRP3 Repeat†** | | | |  |
| HShr8 | + | | | | 7600 | | Absent | |
| HShr9 | + | | | | 12000 | | Absent | |
| HShr14 | + | | | | 9600 | | Absent | |
| HShr17 | + | | | | 240 | | Absent | |
| HShr21 | + | | | | 8800 | Absent | | |
| HShr34 | + | | | | 8000 | Absent | | |
| HShr35 | + | | | | 6000 | Absent | | |
| HShr45 | + | | | | 13200 | **Present** | | |
| HShr56 | - | | | | Neg | Absent | | |
| HShr64 | - | | | | Neg | **Present** | | |
| HShr68 | - | | | | Neg | Absent | | |
| HShr70 | + | | | | 7200 | Absent | | |
| HShr74 | - | | | | Neg | Absent | | |
| HShr76 | - | | | | Neg | **Present** | | |
| HShr79 | + | | | | 440 | Absent | | |
| HShr80 | + | | | | 320 | Absent | | |
| HShr82 | | + | 280 | | | | Absent | |
| HShr87 | | + | 10320 | | | | Absent | |
| HShr100 | | + | 1400 | | | | Absent | |
| HShr102 | | + | 11080 | | | | Absent | |
| HShr107 | | + | 5080 | | | | Absent | |
| HShr109 | | + | 1720 | | | | Absent | |
| HShr113 | | - | Neg | | | | **Present** | |
| HShr122 | | + | 160 | | | | Absent | |
| HShr157 | | + | 12520 | | | | **Present** | |
| HBab1 | | + | 4720 | | | | Absent | |
| HBab09 | | + | 400 | | | | **Present** | |
| HBab21 | | + | 800 | | | | Absent | |
| HBab23 | | + | 9760 | | | | Absent | |
| HBab24 | | + | 14200 | | | | Absent | |
| HBab25 | | + | 10600 | | | | Absent | |
| HBab28 | | + | 28920 | | | | Absent | |
| HBab31 | | + | 11520 | | | | Absent | |
| HBab32 | | + | 11920 | | | | Absent | |
| HBab47 | | + | 12080 | | | | **Present** | |
| HBab48 | | + | 5120 | | | | **Present** | |
| HBab49 | | + | 13800 | | | | **Present** | |
| HBab50 | | + | 8680 | | | | **Present** | |
| HBab63 | | - | Neg | | | | **Present** | |
| HBab64 | | + | 3560 | | | | Absent | |
| HBab66 | | + | 3240 | | | | Absent | |
| HBab68 | | + | 2880 | | | | Absent | |
| HBab75 | | + | 3480 | | | | Absent | |
| HBab80 | | + | 1480 | | | | Absent | |
| HBab85 | | + | Neg | | | | Absent | |
| HBab87 | | + | 160 | | | | Absent | |
| HBab90 | | - | Neg | | | | **Present** | |
| HBab92 | | + | 240 | | | | Absent | |
| HBab94 | | - | Neg | | | | Absent | |
| HBab101 | | + | 200 | | | | Absent | |
| HKum11 | | + | 160 | | | | **Present** | |
| HKum12 | | + | 120 | | | | Absent | |
| HKum14 | | - | Neg | | | | Absent | |
| HKum27 | | - | Neg | | | | Absent | |
| HKum36 | | + | 8280 | | | | **Present** | |
| HKum37 | | + | 7680 | | | | **Present** | |
| HKum38 | | + | 4520 | | | | **Present** | |
| HKum48 | | + | 3560 | | | | **Present** | |
| HKum57 | | - | Neg | | | | **Present** | |
| HKum59 | | + | 11560 | | | | Absent | |
| HKum60 | | + | 11000 | | | | Absent | |
| HKum61 | | + | 10200 | | | | Absent | |
| HKum67 | | - | Neg | | | | Absent | |
| HAss14 | | + | 560 | | | | Absent | |
| HAss51 | | + | 600 | | | | Absent | |
| LShr3 | | - | Neg | | | | Absent | |
| LShr5 | | + | 80 | | | | Absent | |
| LShr44 | | + | 8000 | | | | Absent | |
| LShr132 | | + | 400 | | | | Absent | |
| LShr133 | | + | 200 | | | | **Present** | |
| LShr140 | | + | 200 | | | | Absent | |
| LShr157 | | + | 8000 | | | | Absent | |
| LShr177 | | + | 6800 | | | | Absent | |
| LShr180 | | + | 200 | | | | **Present** | |
| LBab30 | | - | 1200 | | | | Absent | |
| LBab34 | | + | 2800 | | | | Absent | |
| LBab37 | | + | 3200 | | | | Absent | |
| LBab43 | | + | 600 | | | | Absent | |
| LBab67 | | + | 3000 | | | | Absent | |
| LBab82 | | + | 600 | | | | Absent | |
| LBab98 | | + | 1400 | | | | Absent | |
| LKum12 | | - | Neg | | | | **Present** | |
| LKum16 | | - | Neg | | | | **Present** | |
| LKum17 | | - | Neg | | | | **Present** | |
| LKum39 | | + | 600 | | | | Absent | |
| LKum61 | | + | 4000 | | | | Absent | |
| LKum68 | | + | 320 | | | | Absent | |
| LKum74 | | + | 360 | | | | **Present** | |

† (**Present** in yellow bold) =indicate the presence of one or more novel PfHRP3 repeat type in the respective isolates
