# Supplementary material for: Genetic variation of Plasmodium falciparum histidine-rich protein 2 and 3 in Assosa zone, Ethiopia: its impact on the performance of malaria rapid diagnostic tests
Source: Malar J. 2021 Oct 9;20:394. doi: 10.1186/s12936-021-03928-3 (PMC8502267; doi:10.1186/s12936-021-03928-3)
Supplement: Supplementary file 10 — Additional file 10. Effect of the number of PfHRP2 and PfHRP3 amino acid repeat on the performance of PfHRP2 RDT. [file 12936_2021_3928_MOESM10_ESM.docx]

| Additional File 10 : Effect of the number of PfHRP2 and PfHRP3 amino acid repeat on the performance of PfHRP2 RDT | | | | | | |  |
| --- | --- | --- | --- | --- | --- | --- | --- |
| **PfHRP** | **Repeat Type** | **Repeat count** | **PfHRP2 RDT** |  |  |  | |
|  |  |  | Pos | Neg | Total | P-Value⁎ | |
|  | **Type 1** | 1 | 7 | 2 | 9 |  | |
| **PfHRP2** |  | 2 | 12 | 6 | 18 |  | |
| (N=48) |  | 3 | 13 | 4 | 17 | 0.944 | |
|  |  | 4 | 2 | 1 | 3 |  | |
|  |  | 5 | 1 | 0 | 1 |  | |
|  | **Type 2** | 1 | 0 | 1 | 1 |  | |
|  |  | 5 | 0 | 1 | 1 |  | |
|  |  | 6 | 1 | 0 | 1 |  | |
|  |  | 7 | 1 | 0 | 1 | 0.456 | |
|  |  | 8 | 2 | 1 | 3 |  | |
|  |  | 10 | 2 | 2 | 4 |  | |
|  |  | 11 | 4 | 1 | 5 |  | |
|  |  | 12 | 12 | 4 | 16 |  | |
|  |  | 13 | 9 | 1 | 10 |  | |
|  |  | 14 | 3 | 2 | 5 |  | |
|  |  | 15 | 1 | 0 | 1 |  | |
|  | **Type 6** | 0 | 2 | 0 | 2 |  | |
|  |  | 2 | 4 | 3 | 7 |  | |
|  |  | 3 | 16 | 6 | 22 | 0.95 | |
|  |  | 4 | 8 | 3 | 11 |  | |
|  |  | 5 | 3 | 1 | 4 |  | |
|  |  | 6 | 2 | 0 | 2 |  | |
|  | **Type 7** | 3 | 2 | 1 | 3 |  | |
|  |  | 4 | 4 | 1 | 5 |  | |
|  |  | 5 | 5 | 3 | 8 |  | |
|  |  | 6 | 8 | 2 | 10 |  | |
|  |  | 7 | 7 | 2 | 9 | 0.486 | |
|  |  | 8 | 1 | 1 | 2 |  | |
|  |  | 9 | 6 | 1 | 7 |  | |
|  |  | 10 | 0 | 2 | 2 |  | |
|  |  | 13 | 2 | 0 | 2 |  | |
|  | **Type 10** | 0 | 1 | 1 | 2 |  | |
|  |  | 1 | 21 | 10 | 31 | 0.273 | |
|  |  | 2 | 13 | 2 | 15 |  | |
|  |  |  |  |  |  |  | |
| **PfHRP3**  **(N=88)** | **Type 1** | 0 | 4 | 1 | 5 |  | |
|  |  | 1 | 57 | 16 | 73 | 0.928 | |
|  |  | 2 | 4 | 0 | 4 |  | |
|  |  | 3 | 5 | 1 | 6 |  | |
|  | **Type 4** | 0 | 8 | 2 | 10 |  | |
|  |  | 1 | 62 | 16 | 78 | 1.00 | |
|  | **Type 7** | 0 | 2 | 0 | 2 |  | |
|  |  | 1 | 67 | 18 | 85 |  | |
|  |  | 2 | 1 | 0 | 1 | 1.00 | |
|  | **Type 15** | 0 | 3 | 1 | 4 |  | |
|  |  | 1 | 65 | 17 | 82 |  | |
|  |  | 2 | 2 | 0 | 2 | 1.00 | |
|  | **Type 16** | 7 | 1 | 0 | 1 |  | |
|  |  | 8 | 3 | 0 | 3 |  | |
|  |  | 10 | 3 | 0 | 3 |  | |
|  |  | 11 | 11 | 0 |  |  | |
|  |  | 12 | 17 | 12 | 29 | 0.03 | |
|  |  | 13 | 12 | 0 | 12 |  | |
|  |  | 14 | 5 | 1 | 6 |  | |
|  |  | 15 | 4 | 0 | 4 |  | |
|  |  | 16 | 14 | 5 | 19 |  | |
|  | **Type 17** | 1 | 1 | 0 | 1 |  | |
|  |  | 2 | 4 | 0 | 4 |  | |
|  |  | 3 | 2 | 3 | 5 |  | |
|  |  | 4 | 9 | 8 | 17 | 0.00 | |
|  |  | 5 | 13 | 7 | 20 |  | |
|  |  | 6 | 25 | 0 | 25 |  | |
|  |  | 7 | 11 | 0 | 11 |  | |
|  |  | 8 | 5 | 0 | 5 |  | |
|  | **Type 18** | 0 | 1 | 0 | 1 |  | |
|  |  | 1 | 2 | 0 | 2 |  | |
|  |  | 2 | 53 | 11 | 64 | 0.436 | |
|  |  | 3 | 13 | 7 | 20 |  | |
|  |  | 4 | 1 | 0 | 1 |  | |
|  | **Type 20** | 0 | 1 | 0 | 1 |  | |
|  |  | 1 | 63 | 17 | 80 | 1.00 | |
|  |  | 2 | 6 | 1 | 7 |  | |

⁎P-value using Fisher exact test , **N=**Total number of Samples, Pos=Positive, Neg=Negative
